# Non-alcoholic fatty liver disease in patients with type 2 diabetes: diagnostic and therapeutic considerations

**DOI:** 10.1007/s42000-023-00514-x

**Published:** 2023-12-14

**Authors:** Eleni-Maria Paraschou, Almog Shalit, Stavroula A. Paschou

**Affiliations:** Endocrine Unit and Diabetes Center, Department of Clinical Therapeutics, Alexandra Hospital, School of Medicine, National and Kapodistrian University of Athens, 80 Vasilisis Sophias, 11528 Athens, Greece

Non-alcoholic fatty liver disease (NAFLD) refers to a spectrum of chronic liver diseases unrelated to alcohol consumption, including simple steatosis, non-alcoholic steatohepatitis (NASH), advanced liver fibrosis, and cirrhosis [1]. NAFLD affects approximately 25% of the general population. However, the prevalence and complications are often underestimated since most patients have only mild steatosis and do not develop NASH (only 10% of NAFLD patients present with NASH) or liver fibrosis, and the diagnosis is often based on incidental findings (e.g., abnormal liver function tests—LFTs) [2]. It is important to point out that NAFLD usually does not cause symptoms. However, as the disease progresses to NASH, up to 40% of patients develop advanced fibrosis (3). Five percent of these patients develop cirrhosis every year, which may lead to severe hepatic complications such as liver decompensation and hepatocellular carcinoma (HCC, 7.5% occurrence every year in cirrhotic patients) [3].

Recently, the use of the term metabolic dysfunction-associated fatty liver disease (MAFLD) has been proposed as it highlights the coexistence of liver steatosis with metabolic syndrome and other relevant diseases [3]. Indeed, up to 70% of patients with type 2 diabetes mellitus (T2DM) have concomitant NAFLD, and NAFLD is considered a risk factor for development of T2DM. This relationship between T2DM and NAFLD is thought to be mediated by insulin resistance [4]. Other factors that increase the incidence of NAFLD in T2DM patients are an unhealthy lifestyle, smoking, and obesity as well as hyperlipidemia, hypertension, and other cardiovascular diseases (CVD) [5]. NAFLD in T2DM patients is associated with poorer outcomes; in particular, prevalence of advanced liver fibrosis is higher in T2DM patients (12–21%) and these patients are at increased risk of cirrhosis, liver-related events and mortality, and cardiovascular complications [1, 6]. In this regard, screening for NAFLD and advanced fibrosis in T2DM patients has been recommended by major organizations and is included in most recent guidelines [1, 7].

The latest publications underline the importance of screening T2DM patients for NAFLD and identifying those patients at higher risk for advanced fibrosis and progression to cirrhosis [6]. Liver biopsy is the gold standard for the diagnosis and staging of liver disease. However, it is not considered suitable for screening due to its limitations, such as difficulty to apply in large populations, low cost-effectiveness, sampling errors which may lead to mis-staging, and post-procedural complications [6]. As a result, various non-invasive tools have been developed in order to detect patients at high risk for fibrosis. These can be broadly categorized into serum biomarkers (non-invasive tests, NITs) and imaging techniques [3]. NITs can either be indirect markers or direct measures of steatosis, inflammation, or fibrosis. Numerous NITs are currently under evaluation in clinical trials. However, most have not been widely studied in specific populations such as T2DM patients [3]. The latest guidelines from the American Diabetes Association (ADA) and the American Association for the Study of Liver Diseases (AASLD) suggest the use of the fibrosis-4 (FIB-4) index as a first-step risk assessment tool for significant fibrosis [1, 7]. FIB-4 is an indirect biomarker of fibrosis that can easily be applied in diabetic populations as it does not measure glucose-related parameters and has a strong negative predictive value for ruling out low-risk diabetic patients with a cut-off value of 1.3 [7]. Patients with a FIB-4 score between 1.3 and 2.67 are considered at intermediate risk, while a FIB-4 score over 2.67 indicates high risk for advanced fibrosis [7]. In intermediate and high-risk patients, use of additional NITs, such as the enhanced liver fibrosis (ELF) test, is suggested as a second-step risk assessment tool in order to identify patients in need of further hepatology referral [7]. NITs can also be used in combination with imaging tests as follow-up tools and, additionally, as predictive markers for liver-related events in advanced disease [6, 7].

Along the same lines, studies have pointed out that the use of NITs alone as risk assessment tools might lead to both false negative and false positive results which may complicate further investigation and patient referrals [8, 9]. Therefore, inclusion of imaging techniques in the screening of high-risk T2DM patients is recommended [1]. In one study, abdominal ultrasound in all T2DM patients over 50 years old with suggestive clinical features (BMI > 30, abnormal LFTs) is advised [10]. Conventional ultrasound (CUS) is a point-of-care, affordable, and easily available diagnostic tool which has high specificity for the detection of moderate-to-severe steatosis and signs of cirrhosis and can be used as a surveillance tool in cirrhotic patients [11]. However, it is not recommended as a diagnostic tool for NAFLD due to its limitations (e.g., low sensitivity, operator bias, and patients’ BMI) [7]. Vibration-controlled transient elastography (VCTE), which can assess liver fibrosis via liver stiffness measurement (LSM) and liver steatosis with the controlled attenuation parameter (CAP), is the point-of-care imaging method which is recommended by most recent guidelines as a second-step diagnostic tool in patients with a FIB-4 score over 1.3 [1, 7]. Patients with intermediate or high VCTE results require further hepatology referral [7]. VCTE can also be used in combination with LFTs as a follow-up tool [7]. Acoustic radiation-forced impulse imaging (ARFI) and shear wave elastography (SWE) are recently developed tools that can be added to CUS imaging to enhance liver fibrosis quantification and stratify the risk for HCC but still lack evidence of their implementation in everyday practice [3]. Computed tomography is not recommended for repeated imaging due to radiation exposure [10]. Lastly, imaging methods involving magnetic resonance (MR), such as MRI proton density fat fraction (MRI PDFF) for steatosis and MR elastography, have provided optimum quantitative results but are more expensive and are mostly used for clinical trials and specialized patient referrals [11].

The aim of NAFLD/NASH treatment is to prevent or reverse steatosis, inflammation, and fibrosis, if present [12]. Currently, there are no specific approved pharmacological treatments. Management of NAFLD is based on lifestyle interventions and optimizing glucose and lipid levels [1, 6]. A minimum of 5–10% weight loss via diet and/or exercise has been associated with a reduction in liver steatosis, fibrosis regression, better glycemic control, and lipid management [10]. Namely, these lifestyle interventions include mild-intensity aerobic exercise on a weekly basis and dietary modifications based on the Mediterranean and low-carbohydrate diets [10, 13]. Several studies have demonstrated a link between statin use and improved laboratory and histological findings, mostly in NAFLD patients with increased CVD risk; however, it has not proven beneficial in T2DM patients [7, 14]. Optimal selection of antidiabetic agents is important in diabetic patients with NAFLD [1]. Specifically, use of glucagon-like peptide 1 (GLP-1) agonists has provided evidence of histologic improvement, along with significant weight loss, and is therefore recommended [7, 12]. Additionally, pioglitazone, which leads to lipid redistribution via PPAR pathways, has shown improvement both in LFTs and histological parameters [1]. Nonetheless, thiazolidinediones should be used with caution considering such side effects as fluid retention which may lead to congestive heart failure [1]. Other glucose-lowering agents may be continued as clinically indicated, but these therapies lack evidence of benefit in NAFLD, as stated in the ADA guidelines [1]. Bariatric surgery, which is currently recommended in the ADA and AASLD guidelines, can achieve steatohepatitis resolution through increased GLP-1 levels and significant weight loss [7]. However, candidates for bariatric surgery, especially those with cirrhosis, should be referred to multidisciplinary centers in order to minimize complications [7, 10]. Novel medications in phase III trials, such as agonists of additional PPAR pathways, the liver-selective thyroid hormone receptor THR-β agonist resmetirom, and the farsenoid X receptor obeticholic acid, have demonstrated promising results regarding efficacy and safety [14–16].

Overall, it is necessary to screen T2DM patients for NAFLD, especially when relevant comorbidities, such as obesity, are present. Screening for NAFLD must be cost-effective, easy, and safe to apply in primary care settings. The current recommendations include a multistep screening algorithm with NITs and imaging techniques, while further referral to specialized centers is suggested for patients with ambiguous results or advanced NASH and fibrosis. At the moment, no specific treatments are available, and management is based on lifestyle changes and regulation of concomitant cardiometabolic risk factors.
